# Facilitators and barriers to antiretroviral therapy adherence among HIV-positive adolescents living in Tanzania

**DOI:** 10.1186/s12889-021-12323-1

**Published:** 2021-12-13

**Authors:** Cosette Audi, Ola Jahanpour, Gretchen Antelman, Laura Guay, Mastidia Rutaihwa, Roland van de Ven, Godfrey Woelk, Sarah J. Baird

**Affiliations:** 1 Elizabeth Glaser Pediatric Aids Foundation, 1140 Connecticut Ave NW, Suite #200, Washington, DC, 20036 USA; 2 Elizabeth Glaser Pediatric Aids Foundation, Dar es Salaam, Tanzania; 3 National AIDS Control Program, Ministry of Health, Community Development, Gender, Elderly and Children, Dar es Salaam, Tanzania; 4 Department of Global Health, George Washington University, Washington, D.C., USA

**Keywords:** Adolescent, HIV, Adherence, Social support, Qualitative methods

## Abstract

**Background:**

Adolescents living with HIV face substandard outcomes along the continuum of care, including higher rates of poor adherence and virologic failure. Support groups have been identified as a method to improve adherence, but there is insufficient evidence regarding their effectiveness. This study seeks to examine the protective influences for and barriers to antiretroviral therapy (ART) adherence in HIV-positive adolescents living in Tanzania.

**Methods:**

This is a qualitative study conducted in Tanzania from January to March 2018. The sample of adolescents aged 10-19 (*n* = 33) was purposefully selected based on age, gender, and support group attendance to capture a broad range of experiences. Participants completed an in-depth interview, covering topics such as retention in HIV services, support group experiences, and joys and challenges of adolescent life. Interviews were coded and themes related to ART adherence were identified and summarized.

**Results:**

Support groups helped promote adherence by improving adolescents’ knowledge and confidence. Participants associated joining support groups with an improvement in health. Almost every participant described the significant positive influence a treatment supporter had on adherence. Adolescents’ daily schedules and emotional state served as a barrier to adherence. Furthermore, adherence was negatively impacted by participants’ fear of accidental disclosure.

**Conclusion:**

Logistical and psychosocial factors can hinder adherence. Interventions that provide both education and psychosocial support, such as peer support groups, have the potential to improve health outcomes for this population, but may not address more persistent barriers to adherence rooted in lack of treatment support from family members or friends who have not been disclosed to, or lack of transportation funds/food security.

**Supplementary Information:**

The online version contains supplementary material available at 10.1186/s12889-021-12323-1.

## Background

There are 2.1 million adolescents living with HIV, 85% of whom reside in sub-Saharan Africa [1, 2] This number is likely to grow due to the increased survival of perinatally infected individuals, as well as the high rates of HIV incidence among youth in sub-Saharan Africa [1]. Globally, about 32% of new infections occur among adolescents/youth age 10-24 years [3]. The number of adolescents dying from HIV-related causes has also risen. HIV has become the leading cause of adolescent death in Tanzania for 10-14 years-olds and the second leading cause for 15-19 year-olds in Tanzania and globally [4].

Adolescents face substandard outcomes along the continuum of care. As of 2020, from 25 to 80% of adolescents with HIV in sub-Saharan African focus countries were not on antiretroviral therapy (ART) and adolescents have lower viral suppression rates compared to adults [3], and HIV-positive adolescents have demonstrated higher rates of poor adherence and virologic failure than children or adults [2, 5–10]. Estimates of adolescent adherence have ranged widely from 16 to 99%, and there is a lack of high-quality studies examining this topic [11, 12]. Adolescents living with HIV contend with complex psychosocial factors, as well as gaps in knowledge about HIV and its prevention. This population has been found to exhibit increased rates of mental health problems, including depression and suicide attempts, which have been associated with incomplete adherence [13, 14]. In addition to psychosocial challenges, adolescents lack sufficient information about HIV. A survey conducted in countries with generalized epidemics found that less than half of adolescents aged 15-19 years had a basic understanding of HIV [2], and fewer than one-third among this age group in Tanzania [15]. Disparities in knowledge about HIV prevention have been associated with difference in gender, education, household wealth, and place of residence [2].

One potential strategy that has been identified to improve adherence is the use of peer support groups. Support groups have been recommended as a means of improving entry into HIV care and of increasing ART adherence [16]. There is mixed evidence, however, about the effectiveness of support groups in improving adherence [17–21].

The objective of this qualitative study is to examine the protective influences for and barriers to ART adherence in HIV-positive adolescents living in the Arusha and Kilimanjaro regions of Tanzania. Furthermore, this study seeks to build upon the current literature regarding peer support groups [16–20, 22] to determine the perceived effectiveness of such groups in this specific population. This analysis aims to provide insight into the daily lives and experiences of HIV-positive adolescents. A deeper understanding of the day-to-day lives of HIV-positive adolescents is a crucial step for decreasing the number of new infections and improving health outcomes along the continuum of care.

## Methods

The study was conducted in seven health facilities in the Arusha and Kilimanjaro regions of Tanzania from January to March 2018. Each facility hosted a monthly support group for HIV-positive adolescents, facilitated either by a healthcare provider or an adolescent peer in addition to the healthcare provider. A list of eligible participants was generated at each site. In order to be eligible, a participant had to be HIV-positive, 10 to 19 years of age, be reachable by phone, and registered at the participating health facility for at least 1 year. Adolescents were excluded if they did not know their HIV status to prevent unintentional disclosure. Each eligible adolescent was categorized based on age, gender, and club attendance, and participants were then selected from each category ensuring representation from each group at each facility. Younger adolescents were defined as age 10-14, while older adolescents were age 15-19. Club attendance was defined as attending at least one support group meeting in the past twelve months. Adolescents were invited to complete an in-depth interview that lasted approximately one hour. Based on themes identified in the existing literature (summarized in the introduction), which included psychosocial support, knowledge about HIV and impact of peer support groups, a semi-structured interview guide was used to collect information about retention in HIV services, adherence facilitators and barriers, support group experiences, and joys and challenges of adolescent life (Additional file 1). The interview guide built upon these themes and contained questions such as (a) What has your experience with the Adolescent Support groups been like, (b) How do you take your pills/antiretrovirals (ARVs) every day? What or who helps you remember to take them, (c) Do you have a treatment supporter? If so, can you tell me about this person and how they help you with your HIV care, (d) Which members of your family know your status, and (e) What do you envision for your future, such as your work, plans for marriage and family. Interviews were conducted in Swahili by trained research coordinators and were audio recorded.

### Data analysis

All interviews were simultaneously transcribed and translated into English. A codebook was generated through an iterative process. The initial codebook was developed based on the interview guide and preliminary data and revised as new themes were identified (Additional file 2). The transcripts were then coded using MaxQDA software (version 12.3.3). Fifteen percent of the transcripts were coded by two coders to ensure reliability. Any discrepancies were discussed and resolved. Coding reports were generated, and memos were created to summarize the key themes of each code. The analysis presented here focuses on the themes related to ART adherence identified through the interviews related to adherence facilitators and barriers, including adolescent support groups.

## Results

A total of thirty-three adolescents from the seven facilities were interviewed (per facility range 3-9). Seventeen participants were female while sixteen were male. Fourteen interviewees were classified as younger adolescents (age 10-14) and nineteen were older adolescents (age 15-19). Sixteen adolescents had attended a support group meeting in the past twelve months, with four having attended more than one meeting. Seventeen adolescents had not attended a group meeting in the past 12 months.

A number of themes related to ART adherence, including adherence facilitators and barriers were identified through the interviews (Table 1).

**Table 1:** Facilitators for and barriers to ART adherence among HIV-positive adolescents

Domain	Facilitators	Barriers
Adolescent support groups	• Support groups helped adolescents feel more confident and more comfortable taking their medication in front of others. The groups eased feelings of stigma and isolation. • Support groups taught adolescents about HIV and the effects of skipping medication.	• Support groups were not accessible to all adolescents.
Treatment supporters	• Treatment supporters reminded adolescents to take their ARTs. • Supporters provided both logistic and emotional support to adolescents. • Adolescents agreed that health providers respected them, and could not recount any mistreatment from providers	• Not all adolescents had access to support networks.
Logistic and Physical	• Adolescents developed routines to ensure proper adherence. • Feeling healthy motivated adolescents to continue taking their medication.	• Changes to daily schedules, such as traveling or running late for school, impacted adherence. • Cost-related barriers, including inability to afford food or transport to the clinic, negatively impacted adherence. • Some adolescents were deterred by ART’s physical side effects. • Fear of disclosing their status often led adolescents to delay taking their medication. • Adolescents developed strategies to hide their medication.
Knowledge and psychosocial	• Adolescents believed that understanding the reasons for medication made adherence easier.	• Late disclosure of HIV status to the adolescent; and fear of disclosing to others • Lack of education about HIV • Anger/despair about HIV status

### Adolescent support groups

Adolescent support groups helped promote adherence by instilling participants of both genders and age groups with knowledge and confidence. Twenty adolescents described the impact such groups had on their attitudes and behaviors. Adolescents who attended support groups reported that meetings provided space to share information and ideas, as well as advice about adherence and living with HIV. They also learned valuable information about ART, including why they are taking medication, the importance of proper adherence, and the effects of skipping medication. Adolescents were taught about HIV and sexual reproductive health and felt that the groups afforded them people to go to with their health questions while empowering them to teach others about HIV. In response to a question about knowledge gained at support groups, a female group attendee, age 15-19 responded *“It has made me strong and confident because some people ask what do you take pills?, so you tell them that these are my immunity and can go ahead and educate them more about HIV”.*

Support groups helped adolescents feel happier, more self-reliant and confident. Being a group member gave them a sense of pride and strength to stay away from bad influences. They were encouraged by other group members to overcome shame or fear. While some participants reported feeling afraid when they began attending clubs, they quickly became more comfortable interacting with their peers. Support groups also helped ease feelings of stigma and isolation. One male group attendee, age 15-19 reported *“Yes information I get here about not isolating myself and to have freedom has helped me to improve myself. I have learned that I should not isolate myself. I also learned that to have this disease doesn’t mean that I don’t have freedom for doing things I am capable of doing****.”***

Adolescents reported changes in behavior and adherence due to support group participation. Adolescents associated joining the support group with an improvement in health. One adolescent reported now feeling comfortable taking medication in front of others, while another participant said they were more confident in their ability to navigate sexual interactions and negotiate condom use. A female group attendee, age 10-14 associated her support group attendance with an improvement with adherence, saying *“Yes, absolutely because before attending the support group, my adherence to medication was very poor. I was not taking my pills on time. I often missed take my medication timely and sometimes miss them completely. But now is different. I know what it means to me to take my pills daily.”*

### Treatment supporters

Almost every participant discussed the role of a treatment supporter in their healthcare. These supporters were influential in the day-to-day lives of male and female adolescents in both age groups. Treatment supporters were typically members of an adolescent’s immediate (mother, father, sibling, or husband) or extended (grandparent, aunt, or uncle) family, but also could be another community member (school matron or friend).

Supporters assisted adolescents in a variety of ways. While some participants were able to take drugs or attend clinic appointments on their own, most relied on treatment supporters. Adolescents reported that treatment supporters reminded them to take ART, provided medical advice, encouraged clinic attendance, and stored their medication in a discrete location. Additionally, supporters helped adolescents pick up their medication from the clinic, especially when adolescents were sick. Treatment supporters also encouraged support group attendance and reminded adolescents about group meetings. Supporters often provided money for transportation to the clinic and healthy food. A male non-group attendee, age 10-14 described the role his mother served as his treatment supporter, saying *“She encourages me not to skip my treatment schedule and explains that if I skip, my health condition will deteriorate. She gives me food on time so as I can take my drugs on time as well; and even if there is no food in the house she will do all she can so as I can get some. She is always insisting that I don’t skip my treatment schedule.”* Several adolescents reported that their treatment supporter was also HIV-positive. In these situations, there was often a bi-directional system of support, with the adolescent and supporter helping each other remember to adhere to medication and attend clinic appointments.

Treatment supporters and extended family/relatives also provided adolescents with emotional support, encouragement and advice. A female non-group attendee (15-19 years) described how her supporters helped her cope: *… sometimes you might … have a bad day which turns you toward deep thinking and frustration, but our treatment supporters never leave us alone … they will see what they can do to help us out, either by consoling us or encouraging us to not to have feelings of being wasted or something like that.*

### Logistic and physical facilitators and barriers

Adolescents’ daily schedule impacted their adherence and having routines and strategies to ensure they took their medication was important. While some remembered on their own, others were prompted by family members and friends or used an alarm. Adolescents often thought to bring extra medication with them while traveling and knew to take their pills even if food is not available. Some felt comfortable taking medication in front of people, while others preferred to wait until they were alone.

Basic socioeconomic barriers such as lack of food or access to transport were not uncommon. Some participants delayed refilling their ART because of being unable to afford transportation to the clinic. One male group-attending adolescent (15-19 years old) described reaching out to a relative for food in order to take his ARVs: *Sometimes it happens that at home we don’t have anything to eat; I can go to my Aunt requesting for her assistance asking for food, explaining her that I have nothing to eat at home and I have to take my tablets. She goes and buys some food for me.*

Unexpected or uncontrollable changes to adolescents’ routines created barriers to adherence. Adolescents discussed being less likely to take their medication if they were not home at the time when they were to take medication or if they were busy with other tasks, such as chores or schoolwork. Some reported that their school schedule interfered with taking medication and made it more difficult to attend clinic appointments or support group meetings.

While many adolescents report preparing for travel by informing their providers, taking extra pills with them or bringing their clinic card with them, some adolescents reported that traveling can make adherence difficult because they run out of medication or miss doses due to lack of privacy or an irregular schedule. Finally, adolescents discussed simply forgetting. This was the most common reason reported for improper adherence in younger adolescents. Adolescents forgot if they did not have a treatment support to remind them, or if they were busy with other things.

Participants reported that their adherence was impacted by how they felt physically. First, participants were motivated by feeling healthy. Adolescents were concerned about feeling ill, developing opportunistic infections, or dying. Some adolescents experienced side effects, including headaches, dizziness, and skin rashes, if they did not properly adhere to ART. Others, however, reported that their adherence may be impacted by the physical side effects of ART, including fatigue, dizziness, nausea, vomiting, headaches, and difficulty focusing. These side effects can interfere with participants’ daily lives and impact their performance in school. One female non-group, age 15-19 adolescent discussed being in a cycle of adherence and non-adherence, saying *“In the beginning when I used to take my pills and feel sleepy, I say to myself let me see what will happen if I don’t take my pills today. I will wake up feeling good the next morning and I therefore say to myself there is no need for taking these pills again. Some other time I will experience very serious cough till I feel like I am dying, so I will them start taking my pills again and when I start feeling ok I will skip taking my pills again.”*

### Knowledge and psychosocial facilitators and barriers

Several adolescents discussed initially not understanding why they were taking ART and were therefore less likely to adhere. Adolescents said that their adherence has improved as they’ve become more knowledgeable about HIV. Understanding the reason why they take medication and the potential impacts of the disease has facilitated proper adherence. When asked about how his adherence had changed, one male group attendee, age 15-19 responded *“In the past I did not have much information and knowledge on how to use the drugs but now I have a lot of knowledge and information and that drugs help me to survive.”*

One adolescent described feeling afraid and isolated after being diagnosed with HIV, while others discussed feeling angry. One participant described how his diagnosis feels unfair and that he at times just gets tired of taking medication. A female non-group attendee, age 15-19, lamented *“In most times it is out of despair … It is just that I feel why is it only me who is taking the drugs; I don’t see any of my relative taking the drugs, I don’t see my Dad or others taking the drugs, it is only me; I know my siblings were also tested and found negative; now why is it only me? That is what bothers me the most.”*

Another significant factor impacting adolescent adherence was disclosure. While adolescents reported that a variety of people knew their HIV status including family, and other community members, the actual act of disclosure was often done by another person, including an adolescent’s mother, grandparent, or health care provider. Several adolescents reported never having personally disclosed to anyone and struggled with the idea of having to disclose to others.

Adolescents provided a variety of reasons for choosing not to disclose their HIV status. First, adolescents were concerned about how people would react. They worried about experiencing discrimination or isolation. Additionally, they were concerned that the people they disclosed to would tell others, making them a target of rumors and gossip. Adolescents were concerned about being the only person with HIV at their school and felt as if there was no one they could trust. Several adolescents reported being told not to share their status with anybody. A female group attendee, age 15-19 explained *“My sister warned me about that. She told me I must keep that secret, otherwise if other students will know about my status they might start pointing fingers to me, disgrace me. She told me so that I won’t be humiliated by other students.”* This reason was frequently shared by adolescents in the younger age group. One participant said that they would not even know how to disclose. Finally, several older participants did not see any value in disclosing their status, arguing that they managed their HIV care on their own and did not need anyone else to help them. A male group attendee, age 15-19 argued, *“I don’t think if there is any importance of disclosing about my status to my family because I am now grown up and understand the importance of my medications.”*

Participants discussed challenges they faced keeping their status private, from both their inner social circles and the broader community. Adolescents described having to constantly try to act “normally” to prevent people from noticing their illness. The most common strategies mentioned to avoid detection were storing pills in a discrete location and taking medication in private. Adolescents hid their ART in their rooms, bags, or trunks at school. They also commonly transferred ART into discrete packaging. A female non-group attendee, age 15-19, explained: *“The challenges I was facing before disclosing to my husband was hiding my medications from him. I had to make pockets in all of my skirts where I could hide my pills so that when I was done eating I could take them. In some days when he was at home I didn’t leave my medications anywhere around for him to find them.”*

Participants described extra steps taken to ensure they are not seen taking medication, including sitting alone on the bus, hiding pills in dress pockets, and wrapping medication in paper. Adolescents also reported lying to others in order to avoid disclosing. Adolescents described creating excuses to go home to take medication and to get permission to go to the clinic. Several participants discussed lying about the reason for their medication and their illness, often claiming to have chest problems or another disease. Older adolescents reported lying more often.

Most adolescents who had disclosed their status did not report experiencing any social harm or discrimination because of disclosure. While one participant described that his friends laughed at his disclosure, assuming he was joking about his status, many other reported that they were still accepted by friends, teachers, and family after disclosing. Others said that once family members knew about their HIV status, the family members would remind them to take their medication and encourage them to adhere to treatment.

## Discussion

This analysis is among the first qualitative studies to specifically examine the facilitators and barriers to ART adherence in HIV-positive adolescents living in Tanzania [23]. Increased knowledge about HIV and support for treatment emerged as two primary themes positively influencing adherence. Education about HIV helped adolescents better understand the purpose of medication and the consequences of improper adherence. Support networks composed of peers, healthcare workers, and family members provided psychosocial and financial benefits, in addition to day-to-day assistance with adherence. These findings align with the conclusions of previous studies conducted in similar populations [20, 23–27]. Adolescent support groups combine both knowledge and support by giving young people living with HIV an opportunity to simultaneously learn more about their disease while developing strong social networks.

Participants identified clear impediments to proper ART adherence. Daily life activities, such as school, social events and travel often prevented adolescents from taking their medication at the correct time. Furthermore, adolescents simply forgot, especially if they did not have a trusted treatment supporter to remind them. Some reported lack of funds for transport to the clinic, or food insecurity negatively affected their adherence. Fear of revealing HIV status and the subsequent risk of stigma and isolation also impacted how adolescents took their ART. Although adolescents developed strategies to store and take pills without detection, they described skipping doses if they could not take their medication in private. These data corroborate other studies investigating barriers to adolescent adherence [27–31]. It is worth noting that identified facilitators and barriers to ART adherence were similar between adolescents who attended support groups and those who did not.

The barriers to proper adherence identified in this study reinforce the importance of providing comprehensive care that responds to an adolescent’s holistic needs, not just their HIV status. In addition to clinical care, psychosocial support services and other forms of social protection like nutrition support, transportation subsidies, income generation activities, and health education could help minimize barriers to ART adherence [32–35]. Furthermore, the positive effects of support groups on adolescents’ adherence demonstrates a need to ensure these groups are easily accessible and designed with adolescents’ needs in mind.

This study has several limitations. The study population consists of adolescents who are already engaged in HIV care and who could be contacted by the health facility. Additionally, the study contains only participants who are aware of their HIV-status. These factors suggest that adherence facilitators and barriers reported in this study may not be generalizable to the entire HIV-positive adolescent population, but rather represent a particular lived experience. Third, the interview guide did not explore in detail the impacts on adherence of not accessing adolescent support groups and of not having treatment supporters, limiting the discussion of barriers in these two domains. Finally, given budget and time constraints, our sample size was limited to 33, which did reach saturation on many key topics, but limited analysis on others.

## Conclusion

This study demonstrates that basic and continual education on HIV and ARVs, plus robust support systems can improve ART adherence for adolescents living with HIV in Tanzania. Thus, interventions that provide both education and support, such as support groups, have the potential to improve health outcomes for this population. Lack of education, an absence of support networks, fears of stigma and disclosure, and several logistical factors were prominent barriers, sometimes hindering adherence even among those with psychosocial support. These findings suggest that further research may focus on how shifts in routine and life stage impact adolescents’ ability to adhere, and further examine the role played by support networks, including the relationship between an adolescent and their primary treatment supporter and broader social network of family and community, especially in light of managing fears or stigma and disclosure. Additionally, further research is needed to better understand the experiences of adolescents who are not engaged in HIV care.

## Supplementary Information

**Additional file 1.** Semi-Structured Qualitative Interview Guide. This interview guide was used to conduct the semi-structured interviews with study participants.

**Additional file 2.** Codebook. This codebook was used to analyze the interviews.

## Data Availability

The semi-structured interview guide and the codebook used to collect and analyze the qualitative interviews are included as additional files. The full transcripts of the qualitative interviews are unavailable due to concerns over identifiable data. Questions on these transcripts can be submitted to the authors.

## Abbreviations

ART: Antiretroviral therapy
ARVs: Antiretrovirals
