# Supplementary material for: Facilitators and barriers to antiretroviral therapy adherence among HIV-positive adolescents living in Tanzania
Source: BMC Public Health. 2021 Dec 13;21:2274. doi: 10.1186/s12889-021-12323-1 (PMC8670050; doi:10.1186/s12889-021-12323-1)
Supplement: Supplementary file 1 — Additional file 1. Semi-Structured Qualitative Interview Guide. This interview guide was used to conduct the semi-structured interviews with study participants. [file 12889_2021_12323_MOESM1_ESM.docx]

**Additional File 1:** Semi-Structured Qualitative Interview Guide

- **Questions for those who attended Ariel Club *(facilitated by healthcare worker)***

1. What has your experience with the Ariel Club been like?
   1. What is your favorite part about Ariel Club meetings?
   2. What do you dislike about Ariel Club meetings? What would you change?
   3. Has anyone – family, friends, health workers – encouraged you to attend club meetings?
   4. Has anyone discouraged you?
2. What kinds of things do you talk about during your club meetings?
   1. Probes: Facts about HIV, adherence to medication, disclosure, social challenges, sexual reproductive health, etc.
   2. What do you feel is the most important topic you talk about?
   3. What topics do you wish could be discussed or discussed in more detail, but are not?
3. Can you describe how the information you have received has helped you to improve yourself, or feel better about something, or overcome any challenge….?
   1. Probe: We understand there are big differences between boys and girls, and developmental stages (growth) during adolescence. What are your experiences within your peer group regarding how people look at you, and perceive you?
4. What are your feelings about the other members of the Ariel Club?
   1. Do you feel comfortable sharing with other members of the group? Why or why not?
   2. Do you feel supported by members of the group? Why or why not?
   3. Are you older, younger, or the same age as most members of the group? How do you feel about this?
   4. How could Ariel Clubs be improved?
   5. What would you do to encourage more adolescents to participate?
5. What clinical services do you usually receive when you attend Ariel Club meetings?
   1. If you miss an Ariel Club meeting, how does this affect your medication supply? (Probe: Will you be able to collect your ARVs on another day without missing classes or other duties)
   2. Do you prefer to see the clinician on the same day as the Ariel Club, or come on a separate day for services?
6. Has attending a support group affected your adherence? How so?
7. Can you describe a time when a group member, peer or health provider didn’t understand you, or your needs, or didn’t respect you?
8. Please describe any other challenges you face in attending Ariel Club meetings every month? Probe: transport, food, permissions, other duties, health status.
   1. What would cause you to miss an Ariel Club meeting?
   2. What helps you attend support group meetings?

- **Questions for those who have not engaged in any Ariel Club activities**

1. Are you aware of the Ariel Club at this facility? If yes, what has prevented you from attending any activities?
2. Would you be interested in participating in group meetings or activities where other young people living with HIV meet to discuss their experiences? Why or why not?
   1. Would you feel comfortable disclosing your status and sharing your experiences with other adolescents in the group?
   2. Do you think your parents would support you if you decided to attend Ariel Club activities?
   3. Would you have time to attend Ariel Club meetings?
   4. What would make you more likely to participate?
3. Do any of your friends participate in the Club’s activities?
   1. What have you heard about their experiences with the group?
4. Can you describe a time when a peer or health provider didn’t understand you, or your needs, or didn’t respect you?

- **Questions for those who attended enhanced Adolescent Support Group *(facilitated by healthcare worker and peer)***

1. What has your experience with the Adolescent Support Group been like?
   1. What is your favorite part about Support Group meetings?
   2. What do you dislike about group meetings? What would you change?
   3. Has anyone – family, friends, health workers – encouraged you to attend Adolescent Support Group meetings?
   4. Has anyone discouraged you?
   5. How long have you been attending any type of support group meeting at this facility?
      1. **If respondent has been attending any support group meetings at this facility for 3 years or longer, ask**: How would you describe the difference between the Ariel Club meetings and the enhanced adolescent support group meetings?
2. What kinds of things do you talk about during your Adolescent Support Group meetings?
   1. Probes: Facts about HIV, adherence to medication, disclosure, social challenges, sexual reproductive health, etc
   2. What do you feel is the most important topic you talk about?
   3. What topics do you wish could be discussed or discussed in more detail, but are not?
3. Can you describe how the information you have received has helped you to improve yourself, or feel better about something, or overcome any challenge….?
   1. Probe: We understand there are big differences between boys and girls, and developmental stages (growth) during adolescence. What are your experiences within your peer group regarding how people look at you, and perceive you?
4. Have you been trained as a peer facilitator?
   1. (If trained as a peer)
      1. What have your experiences been like?
      2. Can you describe your activities and experiences organizing community activities, reaching out to other adolescents?
      3. How do you feel about the training you went through to become a peer facilitator? Is there any topic you wish you had learned more about?
   2. (If not trained as a peer)
      1. What do you think the peer facilitators do well? How do you think they could improve?
5. What are your feelings about the other members of the Adolescent Support Groups?
   1. Do you feel comfortable sharing with the other members of the group? Why or why not?
   2. Do you feel supported by members of the group? Why or why not?
   3. Are you older, younger, or the same age as most members of the group? How do you feel about this?
6. What clinical services do you usually receive when you attend support group meetings?
   1. If you miss a support group meeting, how does this affect your medication supply?
   2. Do you prefer to see the clinician on the same day as the support group, or come on a separate day for services?
7. Has attending a support group affected your adherence? How so?
8. Can you describe a time when a group member, peer or health provider didn’t understand you, or your needs, or didn’t respect you?
9. Please describe any other challenges you face in attending Adolescent Support Group meetings every month? Probe: transport, food, permissions, other duties, health status.
   1. What would cause you to miss a support group meeting?
   2. What helps you attend support group meetings?

- **Questions for those who have not engaged in any ASG activities**

1. Are you familiar with the Adolescent Support Group? If yes, what has prevented you from attending any activities?
2. Would you be interested in participating in group meetings or activities where other young people living with HIV meet to discuss their experiences? Why or why not?
   1. Would you feel comfortable disclosing your status and sharing your experiences with other adolescents in the group?
      1. Do you think your parents would support you if you decided to attend ASG activities?
      2. Would you have time to attend support group meetings?
      3. What would make you more likely to participate?
3. Do any of your friends participate in the support group’s activities?
   1. What have you heard about their experiences with the group?
4. Can you describe a time when a group member, peer or health provider didn’t understand you, or your needs, or didn’t respect you?

- **Adherence Barriers and Facilitators (ask to all)**

1. How do you take your pills/ARVs every day? What or who helps you remember to take them?
   1. Probes: Do you take them at the same time every day? When? What/who reminds you to take your drugs? Does anyone else you know have to take drugs regularly so you remind each other?
2. How old were you when you began taking your medication on your own?
3. When you plan to take your ARVs every day, on time, but don’t end up taking them, why does this happen?
   1. Probes: Do you have a harder/easier time on some days, such as school days or weekends? What do you do when you have to travel?
4. Do you ever refuse to take your pills? Why?
   1. Probes: difficulty swallowing the pills, dislike taste, side effects, don’t want to take drugs in front of others, don’t want to be on treatment because its lifelong, feel healthy or some other reason.
5. Have you ever had difficulty getting your medication from the clinic? If yes, why? Has this ever caused you to run out of your pills?
   1. Probe: Distance to clinic, financial barriers, clinic hours, wait times, feeling uncomfortable or unwelcome at the clinic
6. Have you had any side effects or other problems from the medications?
   1. Probes: nausea, diarrhea, headaches, fatigue, hospitalizations
   2. What do you do when you have these problems?

- **Retention in Services**

1. How do you get to the clinic for your appointments? Who accompanies you to your appointments?
   1. Do you face problems getting out of school for clinic appointments? If so, please tell me about that.
   2. What would cause you to miss your appointment?
   3. What helps you attend your appointments?
2. Do you have a treatment supporter? If so, can you tell me about this person and how they help you with your HIV care?
   1. Do you get treatment for HIV or another condition somewhere other than the health facility? If so, where or from whom (e.g., traditional healer)?

- **Challenges at home and school**

1. Do you feel supported by the members of your family?
   1. How do they support you?
   2. Probes: Providing emotional support, helping with medication adherence, attending doctor’s appointments, encouraging involvement with support groups, etc.
2. Which members of your family know your status?
   1. How did you disclose to these family members? What made you decide to disclose?
      1. How did they respond when you disclosed your status?
      2. What helped make this disclosure easier?
   2. If not all members, what challenges do you face keeping your status a secret?
      1. Probe: hiding medication, hiding doctor’s appointments, etc
      2. Why haven’t you disclosed your status to all members of your family? What would help make disclosure easier?
3. What is said about HIV at school?
   1. What challenges have you faced at school related to HIV?
      1. Probes: Missing classes for doctor’s appointments, needing to take medication during the school day, hiding HIV status from classmates, stigma due to HIV status, difficulties due to physical symptoms of HIV, etc.
   2. Have you disclosed your status to any of your classmates?
      1. If yes, what made you decide to disclose? What helped make disclosure easier? How did they react?
      2. If no, why not? What would help make disclosure easier?
   3. Do you feel supported by your classmates? Do you feel supported by your teachers?
4. Is there anyone else you’ve disclosed to? How did they react? What made you decide to disclose?
5. Has being HIV+ impacted your ability to be in an intimate or romantic relationship?
   1. If yes, in what way?
   2. Would you disclose your status to your partner?
   3. Do you support groups or health facilities provide information about sexual reproductive health or contraception?
6. What is said about HIV in the community?
   1. How do you feel around adult community members? Probe: How do you think you are perceived?
   2. Have you experienced any mistreatment? How did you cope? Who or what helped you handle it?
   3. Is there anyone in the community, besides family and friends, that helps support you?
7. Imagine that you have a friend who is experiencing a very scary health issue, such as getting an STI, sexual abuse, unwanted pregnancy, HIV diagnosis? How would you advise them (probe for which type of health issue the friend has had)? Describe. (What could they have done to help themselves? Where did they get support?)

- **Joys and challenges of life**

1. What are some of the things in your life that you feel are going very well now, things that cause you to feel hopeful or happy?
   1. Probes: friends/family, health, school, plans for the future.
2. What are some of the biggest issues (challenges) that you or your closest friends are facing that cause you to feel pressured, confused or unhappy?
   1. Probes: misunderstanding (miscommunication) with family or friends, not having money for important or necessary things, pressure from boys or girls to do join bad groups.
3. What concerns do you have about your future?
   1. Probes: Ability to live a healthy life, ability to achieve goals, potential relationships, ability to have children, etc.
4. What do you envision for your future, such as your work, plans for marriage and family?
