# Supplementary material for: Facilitators and barriers to antiretroviral therapy adherence among HIV-positive adolescents living in Tanzania
Source: BMC Public Health. 2021 Dec 13;21:2274. doi: 10.1186/s12889-021-12323-1 (PMC8670050; doi:10.1186/s12889-021-12323-1)
Supplement: Supplementary file 2 — Additional file 2. Codebook. This codebook was used to analyze the interviews. [file 12889_2021_12323_MOESM2_ESM.docx]

**Additional File 2:** Codebook

This list of codes was used to analyze the in-depth interviews.

| **Code** | **Definition** |
| --- | --- |
| **1.0 INDIVIDUAL LEVEL** | |
| 1.1 Adolescent health care experiences | This is a response by participants which describes actual experiences, or examples of care received or their experience with providers. For responses to questions like, how did you first come to the clinic, they can be coded here, if applicable. |
| 1.1A Positive experiences | These include examples of supportive health providers, important information received, or other positive experiences. For neutral experiences (e.g., statements such as health provider has never disrespected an adolescent client), use this code. |
| 1.1B Negative experiences | These include any descriptions of when health providers didn’t understand or respect an adolescent or when an adolescent felt judged or stigmatized for seeking SRH or other health services. If a description includes both positive and negative aspects of the experience, code as negative. |
| 1.2 ARV side effects | This code is for any mention of side effects caused by ARVs. It also includes any mention of not experiencing any side effects. |
| 1.3 ARV adherence facilitators | This code is for any mention of what helps adolescents adhere to ART. For example supportive family members, fear of getting sick, good counselors who make them feel supported, etc. This code also includes any neutral statements related to ARV adherence, such as “No side effects” or “Have not refused to take pills”. |
| 1.4 ARV adherence barriers | This code is for any mention of the barriers adolescence face adhering to ART. For example can’t remember, scared to take the meds in front of others, challenges picking up the medication, side effects, etc. |
| 1.5 Adolescent joys | These are things in an adolescents’ life that are going well for him or her or make them feel hopeful or happy. Caregiver or health worker responses on what makes adolescents happy can also be included here. |
| 1.6 Adolescent life challenges | These are responses regarding any issues adolescents are facing that make them unhappy, confused, feel pressure or are detrimental to their well-being, such as poverty. These can be challenges that adolescents report their friends are having or that caregivers/health workers/community members report adolescents they know are facing. Challenges can be occurring presently or in the past. |
| 1.6A Impact of HIV on romantic relationships | This code is for any mention of how HIV has impacted adolescents’ ability to be in an intimate or romantic relationship. Any discussion of disclosure to a romantic partner should be coded to this code. |
| 1.7 Adolescent future concerns | Comments about adolescents’ concerns for the future, including their ability to live a healthy life, have/maintain a relationship, ability to have children, etc. These can be concerns that adolescents report their friends are having or that caregivers/health workers report adolescents they know are facing. If adolescents have no concerns about their future, it should be coded here as well. |
| 1.8 Adolescent aspirations | These are descriptions by adolescents or others about adolescents’ goals for the future – what job they would like to have, goals for getting married and having children, provide for other family members, etc. If adolescents have no future goals, code those responses here. |
| **2.0 Family/social/interpersonal LEVEL** |  |
| 2.1 Disclosure | These are descriptions of how children were disclosed to, when, by whom, etc and their feelings about that experience. These can also be descriptions of adolescents disclosing their status to members of their family or peers. |
| 2.1A Challenges keeping HIV status a secret | This code refers to any challenges adolescents have faced to keep their status a secret from people they have not disclosed to. This includes hiding medication, hiding appointments, making excuses, etc. |
| 2.1B Reasons for not disclosing | This refers to any reasons why adolescents have chosen not to disclose their HIV status (e.g., fear of stigma, told not to disclose by parents, etc.) |
| 2.2 Adolescent support from family and other adults | This are responses by participants about familial support or support from other adults provided to or received by adolescents. Use the main code if none of the sub-codes are applicable. For instance, in the case of descriptions of the support provided to adolescents by single-family households. |
| 2.2A Involvement in health/health services | This includes the ways that parents/caregivers/other adults support adolescents with regards to their health or accessing health services (e.g., serving as treatment supporter, picking up drugs for them, reminding them to take drugs, encouragement to attend clinic or support group). This also includes any ways that parents/ caregivers discourage adolescents from accessing health services (e.g. attending support group). Participants’ thoughts regarding caregiver presence during counseling sessions on HIV, FP, etc or whether or not parents should have to consent for their child’s HIV testing should also be coded here. |
| 2.2B Instrumental support | This type of support includes the provision by family members for adolescents’ food, transport, school fees etc. |
| 2.2C Psychosocial support | This includes less tangible types of support provided by the family or other adult (i.e., teacher) such as the adolescent always knowing a family member is there to support them, family members who provide guidance, educate or listen to adolescents’ concerns/issues, etc. |
| 2.3 Safety/vulnerability of adolescents | These are adolescents’ perceptions about the safety of themselves or other adolescents in the community, their vulnerabilities and whether or not they are/seem protected and safe. |
| 2.4 Adolescent perceptions of adults | These are comments by adolescent participants regarding their perceptions or views of adults in their families and communities. **If an adolescent is asked how they think adults perceive them, this can also be included here.** |
| 2.4A Positive perceptions | These are attitudes in which adolescents perceive adults to be people they can talk to, trust, relate to, etc. More neutral views of attitudes can also be included here. |
| 2.4B Negative perceptions | These are attitudes in which adolescents perceive adults to be people they cannot talk to, trust, relate to, etc. Feelings that adolescents are being judged unfairly or treated inappropriately can also be included here. |
| 2.5 Peer perceptions about/behaviors towards other adolescents | These are comments by adolescent participants describing how they perceive and interact with other adolescents in their communities. Any description of interactions between adolescents that take place at club meetings should be coded to 3.7A. |
| 2.6 Experiences of peer educators leading groups | These are responses by participants which recount events that have occurred within the support group related to peers’ leadership of those groups or events that were a direct result of that leadership. Behaviors that have changed as a result of group attendance should be coded under *Group influence on behavior.* |
| **3.0 Organizational (facility, school)** | |
| 3.1 Peer educator training | These include descriptions of training for peer educators – locations, who provided, topics covered, etc. Any recommendations for additional training or training needs should be coded here as well. This also includes any mention of not being trained as a peer educator. |
| 3.2 Health facility attributes of adolescent services | These are responses by participants that describe various aspects of service delivery at the health facility. If the applicable sub-code cannot be found for a particular facility attribute, use this main code. |
| 3.2A Types of services provided | These are the types of services provided at health facilities, such as support groups, adolescent-specific counselling, adolescent-only days, youth-friendly corner, psychosocial support services (e.g., legal protection, nutrition, education or other learning opportunities, shelter, support groups, economic strengthening. Other responses to be coded here: 1) services specifically provided by the provider being interviewed; 2) services provided as a whole at the facility; 3) a description of services for which patients are referred; 4) services received on the days adolescents attend support group meetings. |
| 3.2B HIV counseling messages | These are responses by participants that describe the information or advice from health providers on HIV-related topics, including drug adherence, side effects, disclosure, etc. |
| 3.2C FP counseling messages | These are responses by participants that describe the information or advice from health providers on FP-related topics, such as the pros and cons of different FP methods, delay of sexual intercourse, pregnancy intentions, etc. |
| 3.3 Retention in HIV services at health facility | These are responses by participants that describe what helps or prevents them from remaining in HIV services. If the applicable sub-code cannot be found, use this code. |
| 3.3A Access to health facility | This code is for anything that affects an adolescent’s ability to access the facility such as long wait time, cost of transport, hours of service, location of facility, interference with school, appointment reminders, etc. |
| 3.3B Use of other health facilities and services | This is for discussion of receiving treatment for HIV or other conditions at somewhere other than the usual health facility. This can include other facilities, traditional healers, etc. |
| 3.4 Attendance at support group | This includes basic information about whether or not the adolescent attended support groups, when attended, etc. |
| 3.5 Barriers to group attendance | This code should be used for adolescents who are **not** currently attending support groups. These are any factors that would make it less likely an adolescent would join a club, including attendance at boarding school, an unsupportive parent, or a lack of awareness about the group. |
| 3.6 Facilitators to group attendance | This code should be used for adolescents who are **not** currently attending support groups. These are any factors that would make it more likely an adolescent would join a club, including support from family or stories or encouragement from friends who are involved in groups/clubs. |
| 3.7 Support group attributes | These are responses by participants that describe various aspects of support groups at Ariel Club and enhanced ASG health facilities. If the applicable sub-code cannot be found for a particular support group attribute, use this main code. |
| 3.7A Interactions with group members | These are descriptions of how group members play or talk together in group, support each other outside of group and the extent to which they feel comfortable around each other. |
| 3.7B Encouraging group attendance | This includes strategies or experiences relayed by adolescents or others as to how they encourage adolescents to attend support groups as well as how they were encouraged to attend support groups themselves. This also includes factors that encourage adolescents to continue attending groups. |
| 3.7C Discouraging group attendance | This code is for any mention of people or things that have discouraged adolescents from attending a support such, such as family members, peers, or health providers. This code also includes neutral statements, such as "No one has discouraged me from attending". |
| 3.7D Missing group meetings | Responses should be coded here if they refer to reasons for missing group meetings, how missing meetings affect ART supply, etc. Responses referring to not missing group meetings should be coded to 3.4 Attendance at Support groups. |
| 3.7E Group influence on behavior and attitudes | This will be descriptions of how support groups have affected adolescents’ behaviors, such as adherence to their HIV treatment, protective sex, etc. This also includes descriptions of how support groups have affected adolescents’ attitudes, such as increased knowledge or confidence. |
| 3.7F Preference to combine with clinical services | These are adolescent participants’ responses as to whether or not they prefer to have their clinic visit on the same day as their support group meeting (as opposed to a different day). |
| 3.7G Ages of members | These are responses that describe the ages of other support group members, whether or not members are close in age to the adolescent participant and any thoughts that participants have about the similarities or differences in ages among their fellow support group members. |
| 3.7H Group topics discussed | These are descriptions by participants about the topics covered in support groups and can include health-related issues such as HIV or FP, coping strategies, issues related to support group members family, school or interpersonal life, etc. |
| 3.7I Gaps in topics covered | These should be topics mentioned by participants that they wish were covered more in support groups and can include topics that peer educators would like to talk more about but don’t feel as if they have the knowledge or background. |
| 3.7J Other group activities | These are responses which describe activities other than discussing issues in support groups that the team engages in, such as sports games or income generation activities. If there are activities that a participant would like the group to undertake, these should also be mentioned here. |
| 3.7K Support group leader impressions | These are comments by participants on their perceptions and views of their support group leader. Do they like this person, find them relatable, wish the leader was more like them in some way (gender, age), think they’re effective, etc. |
| 3.7L Support group venue | These are descriptions of the venue where support groups take place – is the space comfortable, private, convenient, big enough, etc. Recommendations to improve upon the venue should be included under this code. |
| 3.7M Ariel Clubs versus enhanced adolescent support groups | This code should be used for any discussion of the differences or similarities between Ariel clubs and the enhanced adolescent support groups. |
| 3.7N Recommendations for improvement | This code should be used for any recommendations to improve support groups that are not captured under the specific codes above, such as those on the support group leader, topics, venue, etc. Also code responses here that indicate participants have no recommendations/nothing to improve. This code is also used for any aspects of support groups that adolescents dislike. |
| 3.8 Health education in schools | This should include responses which describe the information shared in schools on health topics (e.g., HIV and pregnancy prevention) and any services provided (e.g., condom distribution). Please include here perceptions of this outreach – its acceptability, effectiveness, etc. |
| **4.0 Community/Environmental** | |
| 4.1 Advice to adolescents | These are responses by all participants on the scenario presented about adolescents who become pregnant, get an STI or have another health issue, in one’s family or community. |
| 4.2 Community-level responses to HIV/FP | This includes how community members sensitize adolescents and their parents on HIV and FP any work of adolescent peer educators in the community. This is also for any attitudes held by members of the community related to HIV. |
| 4.3 Challenges faced in school | This is for any challenges adolescents have faced at school related to their HIV status. This may include stigma from classmates or teachers, missing classes for appointments, having to take medication at school, etc. This code can also include mention of not facing any challenges at school. Any mention of what is taught in school about HIV should be coded to 3.8 |
